# *Citrus hystrix* Extracts Protect Human Neuronal Cells against High Glucose-Induced Senescence

**DOI:** 10.3390/ph13100283

**Published:** 2020-09-30

**Authors:** Nattaporn Pattarachotanant, Tewin Tencomnao

**Affiliations:** 1 Department of Clinical Chemistry, Faculty of Allied Health Sciences, Chulalongkorn University, Bangkok 10330, Thailand; nat.ahs11@gmail.com; 2 Age-Related Inflammation and Degeneration Research Unit, Chulalongkorn University, Bangkok 10330, Thailand

**Keywords:** *Citrus hystrix*, neuronal senescence, high glucose, SIRT1, cyclin D1, pcdc2, pRb, SH-SY5Y

## Abstract

*Citrus hystrix* (CH) is a beneficial plant utilized in traditional folk medicine to relieve various health ailments. The antisenescent mechanisms of CH extracts were investigated using human neuroblastoma cells (SH-SY5Y). Phytochemical contents and antioxidant activities of CH extracts were analyzed using a gas chromatograph–mass spectrometer (GC-MS), 2,2-diphenyl-1-picryl-hydrazyl-hydrate (DPPH) assay and 2,2′-azino-bis (3-ethylbenzthiazoline-6-sulphonic acid) (ABTS) assay. Effects of CH extracts on high glucose-induced cytotoxicity, reactive oxygen species (ROS) generation, cell cycle arrest and cell cycle-associated proteins were assessed using a 3-(4,5-Dimethylthiazol-2-yl)-2,5-diphenyltetrazolium bromide tetrazolium (MTT) assay, non-fluorescent 2′, 7′-dichloro-dihydrofluorescein diacetate (H_2_DCFDA) assay, flow cytometer and Western blot. The extracts protected neuronal senescence by inhibiting ROS generation. CH extracts induced cell cycle progression by releasing senescent cells from the G1 phase arrest. As the Western blot confirmed, the mechanism involved in cell cycle progression was associated with the downregulation of cyclin D1, phospho-cell division cycle 2 (pcdc2) and phospho-Retinoblastoma (pRb) proteins. Furthermore, the Western blot showed that extracts increased Surtuin 1 (SIRT1) expression by increasing the phosphorylation of Glyceraldehyde 3-phosphate dehydrogenase (GAPDH). Collectively, CH extracts could protect high glucose-induced human neuronal senescence by inducing cell cycle progression and up-regulation of SIRT1, thus leading to the improvement of the neuronal cell functions.

## 1. Introduction

Hyperglycemia is a key characteristic and risk factor that triggers neuronal damage, leads to encephalopathy and causes the development of Diabetes mellitus (DM)-associated neurodegenerative diseases through the induction of neuronal senescence [1,2,3,4]. The incidence of most neurodegenerative diseases, such as Alzheimer’s (AD) and Parkinson’s disease (PD), has been shown to increase exponentially with advancing hyperglycemia and neuronal senescence. Hyperglycemia results in damage of brain structure and is closely related with the development of cognitive impairment and dementia, thus increasing amyloid beta accumulation [5]. Hyperglycemia can cause neuroinflammation, oxidative stress and cell cycle arrest. Cell cycle arrest is an important process associated with neuronal senescence. In replicatively senescent cells, the cell cycle is arrested at the G1 and/or G2 phase [6,7]. Cell cycle regulation is very complex and there are many proteins required for cell cycle progression.

Retinoblastoma (Rb) is a tumor suppressor that is a major regulator of the G1/S transition. Rb can be phosphorylated by cyclin/cyclin-dependent kinases (CDKs) and forms a complex transcription factor E2F to control cell progression through the G1 phase [8].The ability of phospho-Retinoblastoma (pRb) to control the cell cycle has been attributed to E2F suppression [9].

Moreover, there are many CDKs that control the cell cycle, most prominently among them cdk1 or cell division cycle protein 2 (cdc2). The cdc2 level is high in S and G2 but low in G1. Cdc2 can form a complex with both cyclin A and cyclin B for S phase and G2/M transition control, respectively [10].

Cyclin D1 (G1 cyclin regulatory partner) is an important protein that controls cell cycle progression. Many researches indicated that down-regulation of cyclin D1 causes G1 cell cycle can arrest by reducing Rb phosphorylation [11,12,13,14].

Surtuin 1 (SIRT1) is a nicotinamide dinucleotide (NAD+)-dependent deacetylases. SIRT1 plays an important role in inhibiting cell senescence and extending the lifespan of organisms [15,16,17]. Moreover, it is an essential factor in the regulation of many processes in cells such as DNA repair, chromatin structure, metabolism, inflammation and cancer [18].

*Citrus hystrix* (CH), called kaffir lime, is a useful tropical plant native to Southeast Asia. It is a small perennial plant with a hard trunk, smooth bark, smooth trunk and spiny branches. It has green, aromatic and distinctively shaped double leaves. Its fruit is either a single or a bunch. Fresh fruits are rough and green and yellow after ripened. It is commonly used as a very popular ingredient in many dishes. For traditional medicine, whole fruits are used for treating various inflammatory aliments, fever, headache, bad breath, digestion, flu and sore throats [19]. The previous phytochemical report showed that this plant contains various phytoconstituents such as high phenolic, flavonoid, alkaloid, tannins, glycerolglycolipids, tocopherols and furanocoumarins in several parts of CH, such as leaf, peel and juice [20]. These phytochemical compounds exhibited many advantages, such as antioxidant, antibacterial, antifungal, anticholinesterase, anticancer, cardioprotective and antidiabetic activities [21,22,23].

Phytochemical compounds in CH, including citronellal, citronellol, caryophyllene, nerolidol, phytol, ethyl palmitate, sitosterol and α-terpineol and their beneficial effects have been previously described and prompted this investigation.

Citronellal was effective against several pathogenic bacteria and fungi [24,25,26]. It had the potential to speed up the healing process of *Candida*-infected wounds in a diabetic mouse model [27] and it could decrease the cholesterol level in rats [28]. Furthermore, it had high antioxidant activities [29].

Citronellol is a natural component of citronella oil. It is widely used as an effective mosquito repellent and is used in perfume and beauty products. Specific pharmacological effects for citronellol include antinociceptive activity and anti-inflammatory effects [30]. It provides neuroprotective activity [31], hypotensive and vasorelaxant effects [32].

Caryophyllene is found in numerous essential oils and has a high potential to treat or prevent hepatic injury and neuroinflammation [33,34,35].

Nerolidol is found in essential oils of many types of plants and flowers and is known for various medicinal properties, including neuroprotective effects [36], antimicrobial activities [37], anti-biofilm activity [38], anti-fungal activities [39,40]. 

Phytol is widely used as a food additive and a precursor to phylloquinol (vitamin K), tocopherol (vitamin E) and fatty acid phytyl ester production. In the medicinal field, it exerts an anti-inflammatory effect and redox-protective activity [41].

Ethyl palmitate has anti-inflammatory activities [42].

Sitosterol, a phytosterol (plant sterols), lowers the level of serum low-density lipoprotein cholesterol (LDL-C) and the absorption of intestinal cholesterol [43,44]. It also exerts anti-Alzheimer’s activity [45] and antioxidant activity [46,47]. Additionally, it can prevent glutamate and β-amyloid toxicity [48].

α-Terpineol exhibited anti-hypertension, antiproliferation, anti-inflammation, anti-bacteria and antioxidant. Moreover, it could re-establish insulin sensitivity [49,50,51,52,53].

In this study, gallic acid (GA) was used as a positive control. GA has emerged as a strong antioxidant found in fruits and vegetables. GA showed the anti-aging activity on skin [54,55], anti-senescence accelerated mice [56], anti-dementia [57], anti-diabetes [58,59] and neuroprotective effect [24,60].

The aim of this study was to investigate the mechanism for antisenescent activity of CH extracts on a human neuroblastoma cell line (SH-SY5Y). The advantage may be useful for developing alternative medicines to prevent neuronal senescence.

## 2. Results

### 2.1. Antioxidant Properties and Total Phenolic and Flavonoid Contents

In this study, *Citrus hystrix* peels and leaves were designated as CHP and CHL, respectively. To investigate the free radical scavenging capacities of CHP and CHL, we used a 2,2-diphenyl-1-picryl-hydrazyl-hydrate or DPPH assay, as well as a 2,2′-azino-bis (3-ethylbenzthiazoline-6-sulphonic acid) or ABTS assay. Strong antioxidant activity was found in both CHP and CHL extracts. In CHL extract, we found high phenolic (2134.48 ± 1.06 mg(GA)/g of dry weigh) and flavonoid (2856.15 ± 1.24 mg(QE)/g of dry weigh) contents. Table 1 shows free radical scavenging activity data using a DPPH scavenging assay. In Table 2, we show free radical scavenging activity data using an ABTS scavenging assay. Table 3 shows total phenolic and flavonoid content data.

### 2.2. Phytochemical Constituents of CHP and CHL

**Figure 1:**
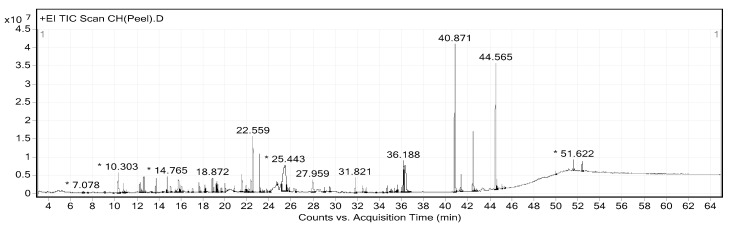
Gas chromatograph–mass spectrometer (GC-MS) chromatogram of Citrus hystrix peels (CHP). * Peaks of proposed phytochemical constituents in CHP were suggested by GC-MS.

**Figure 2:**
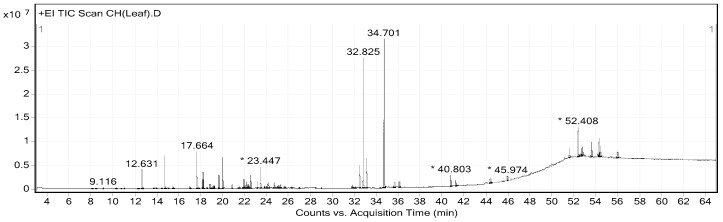
GC-MS chromatogram of *Citrus hystrix* leaves (CHL). * Peaks of proposed phytochemical constituents in CHL were suggested by GC-MS.

The extraction yields of CHP and CHL were 14.17% and 13.04%, respectively. Gas chromatograph–mass spectrometer (GC-MS) analysis showed the presence of different phytochemical compounds in both extracts. All peaks in both CHP and CHL extracts were detected and compared the MS data with databases to identify chromatographic peaks. GC-MS chromatograms of CHP and CHL were shown in Figure 1 and Figure 2. In the percentage of compounds, we identified 18 proposed phytochemical constituents in CHP, which is detailed in Table 4. Moreover, in Table 5, we detail 12 proposed phytochemical constituents in CHL.

### 2.3. Neuronal Senescent Model

**Figure 3:**
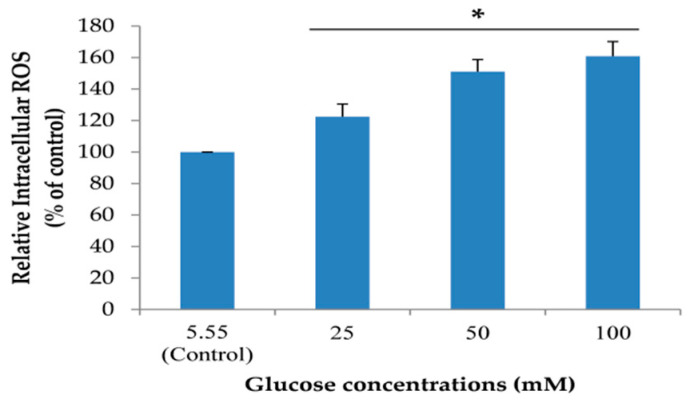
The effect of different concentrations of glucose on intracellular reactive oxygen species (ROS). Relative intracellular ROS level was performed using a microplate reader. Data are mean ± SD, * *p* < 0.05 vs. control. *p* values were 0.03, 0.007 and 0.000 for groups treated with glucose concentrations of 25, 50 and 100 mM, respectively.

**Figure 4:**
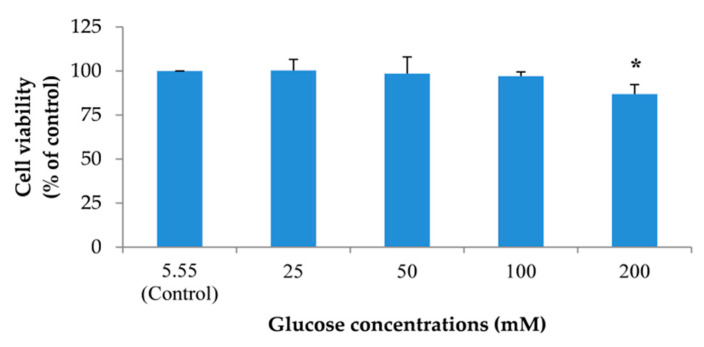
The effect of different concentrations of glucose on SH-SY5Y cell viability. Detection of cell viability was performed using 3-(4,5-Dimethylthiazol-2-yl)-2,5-diphenyltetrazolium bromide tetrazolium (MTT) assay. Data are presented as the means ± SD, * *p* < 0.05 vs. control. *p* values of 200 mM glucose-treated group was 0.000.

To create a neuronal senescent model, SH-SY5Y cells were induced into an expression of intracellular reactive oxygen species (ROS) by treating cells with 5.55 (control) to 100 mM glucose. In addition, the effect of glucose on cell viability was detected. We found that glucose could significantly increase the percentage of intracellular ROS in a dose-dependent manner after treatment for 24 h (Figure 3). Moreover, the highest concentration of glucose that had no effect on cell viability was 100 mM (Figure 4). From all results, we used 100 mM glucose to induce neuronal senescence in this model.

### 2.4. Effects of the Extracts on Cell Viability

**Figure 5:**
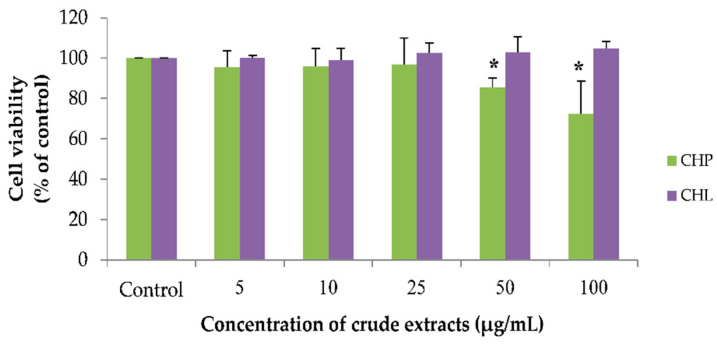
The effect of CHP and CHL on cell viability. Detection of cell viability was performed using an MTT assay. Data are presented as the means ± SD, * *p* < 0.05 vs. control. *p* values were 0.018 and 0.030 in groups treated with CHP concentrations of 50 and 100 µg/mL, respectively.

Using the working concentrations of both extracts, CHP and CHL (0 to 100 µg/mL) were prepared. We found that all concentrations of CHL had no effect on cell viability. For the CHP effect, the results showed that CHP at the concentration of 50 and 100 µg/mL could significantly decrease cell viability. As seen in Figure 5, the percentage of cell viability treated with CHP for 24 h was 85.50 ± 4.56% and 72.48 ± 16.12% in 50 and 100 µg/mL, respectively.

### 2.5. Effects of the Extracts on Intracellular ROS Reduction

**Figure 6:**
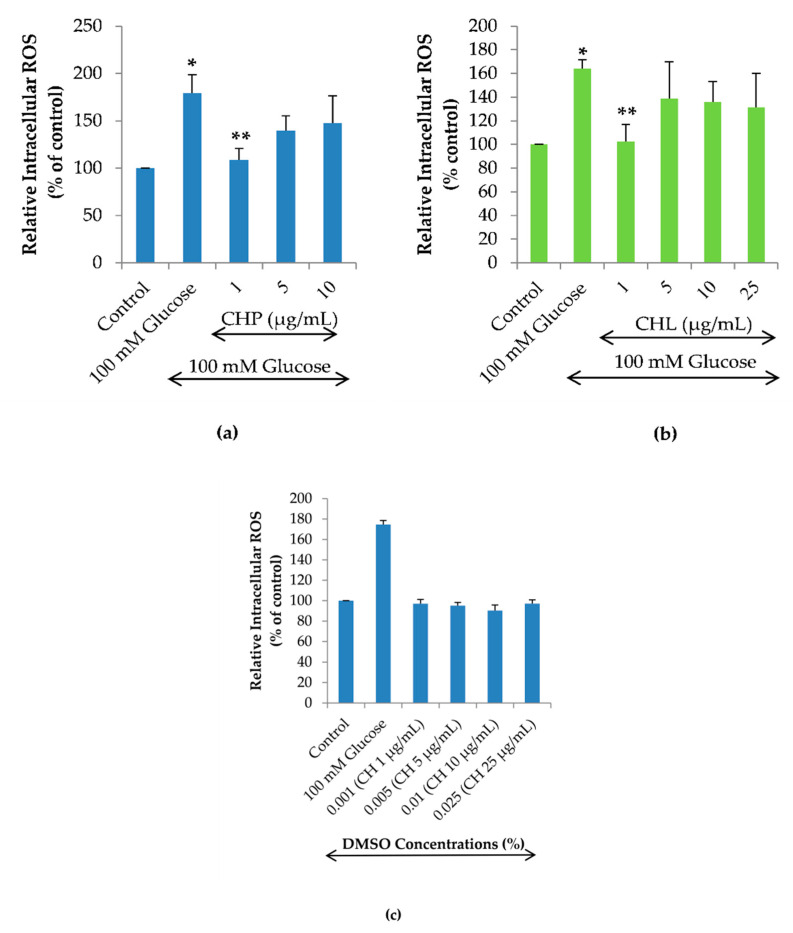
Effects of the extracts on high glucose-induced ROS accumulation in SH-SY5Y cells. Relative intracellular ROS level of SH-SY5Y cells treated with 100 mM glucose alone or combined with different concentrations for either CHP (**a**) or CHL (**b**) for 24 h. The effect of DMSO on relative intracellular ROS level of SH-SY5Y cells treated with (0.001 to 0.025% (*v*/*v*)) DMSO alone (**c**). Data are presented as the means ± SD, * *p* < 0.05 vs. control; ** *p* < 0.05 vs. 100 mM glucose alone. For CHP, *p* value was 0.002 in both 100 mM glucose and 1 µg/mL groups. For CHL, *p* value was 0.000 in both 100 mM glucose and 1 µg/mL groups.

To examine the effects of CHP and CHL on high glucose-induced intracellular ROS, this experiment was investigated by treating cells with 100 mM glucose alone or combined with different concentrations of CHP (1, 5 and 10 µg/mL) and CHL (1, 5, 10 and 25 µg/mL). The dose-response curve of CH extracts was obtained by isovolumetric additions of CHP and CHL solutions of different concentrations so that the DMSO concentration range was from 0.001 to 0.025% (*v*/*v*). We found that both CHP and CHL at 1 µg/mL could significantly reduce the percentage of high glucose-induced intracellular ROS (Figure 6a,b). However, to clarify the effect of DMSO in CH extracts on intracellular ROS accumulation, cells were treated with 0.001 to 0.025% (*v*/*v*) DMSO alone for 24 h. In the H_2_DCFDA assay, we show that the increasing DMSO alone did not affect ROS generation in SH-SY5Y cells (Figure 6c). This result clarified that both CHP and CHL extracts appeared to be effective reducing the intracellular ROS accumulation at the concentration of 1 µg/mL but not at higher concentrations was not caused by DMSO.

### 2.6. Effects of the Extracts on Cell Cycle

**Figure 7:**
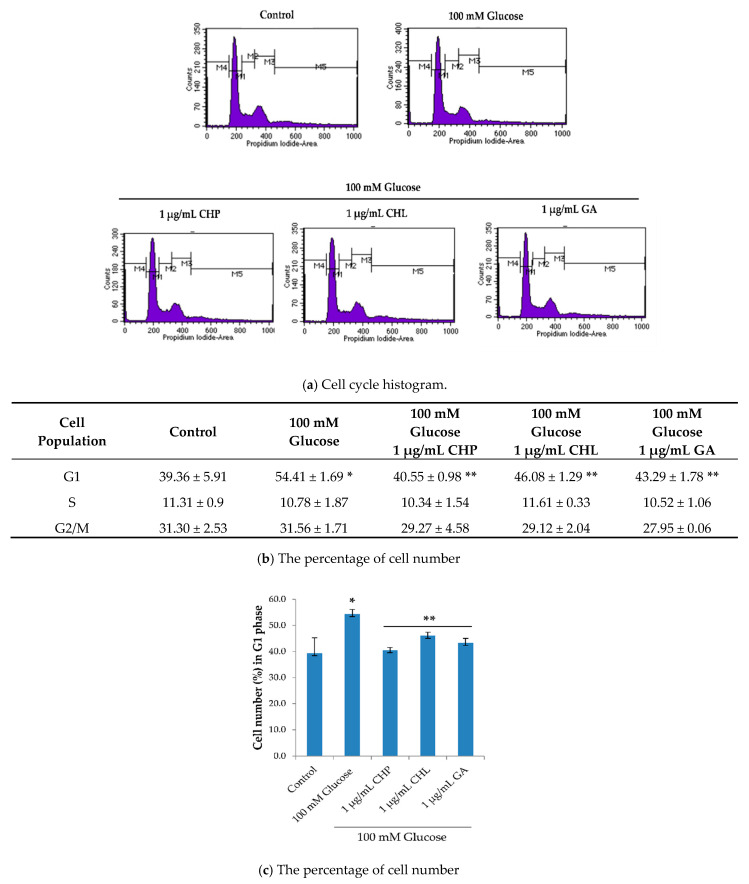
The effect of extracts on the cell cycle. Quantitative determination based on propidium iodide (PI) staining was carried out using a flow cytometer. The results showed (**a**) cell cycle histogram, (**b**,**c**) the percentage of cell numbers. Data are presented as the means ± SD, * *p* < 0.05 vs. control; ** *p* < 0.05 vs. 100 mM glucose alone. *p* values were 0.001, 0.001, 0.039 and 0.007 for 100 mM glucose, CHP, CHL and GA, respectively.

To further understand the ability of both CHP and CHL on neuronal senescence, we determined the activity on cell cycle distribution. Data generated from a flow cytometer demonstrated that the percentage of cells in the G0/G1 phase when treated with 100 mM glucose was significantly higher than in the control group (*p* < 0.05). Moreover, 100 mM glucose arrested cells in the resting phase (G0/G1). The percentage of cells in the G0/G1 phase after treatment combined with 100 mM glucose and 1 µ/mL of either extract or gallic acid was significantly decreased when compared with the group treated with glucose (*p* < 0.05) (Figure 7). Gallic acid (GA) was used as a positive control in this study.

### 2.7. Effect of the Extracts on Cell Cycle-Associated Protein and SIRT1 Expression

**Figure 8:**
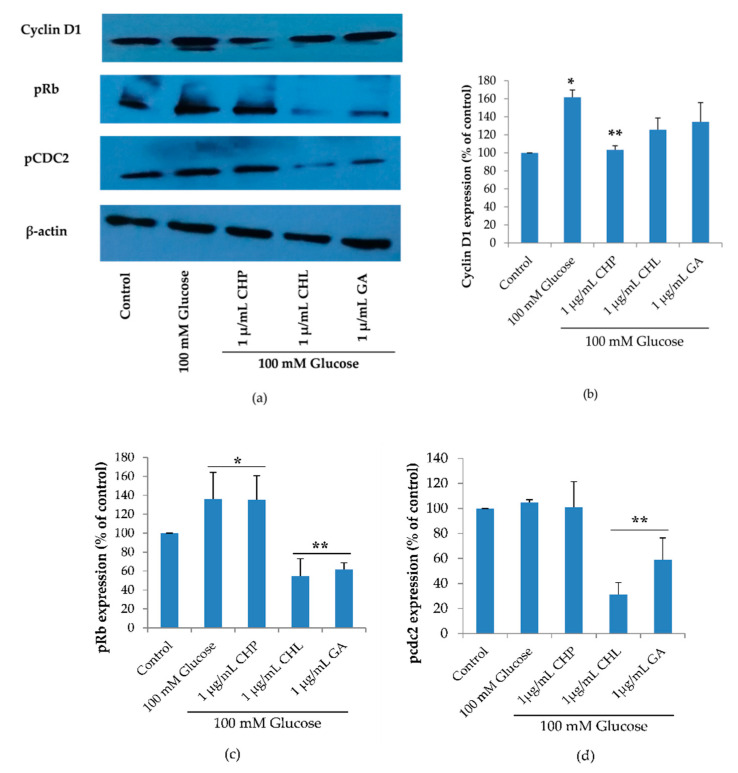
Cyclin D1, phospho-Retinoblastoma (pRb) and phospho-cell division cycle 2 (pcdc2) expression, as shown in a representative Western blot (**a**)**.** Normalized values of Cyclin D, pRb and pcdc2 against β-actin ((**b**)**,** (**c**) and (**d**)**,** respectively). The mean ± SD values of normalized Cyclin D, pRb and pcdc2 expression were obtained from three independent experiments, * *p* < 0.05 vs. control; ** *p* < 0.05 vs. 100 mM glucose alone. For Cyclin D1, *p* values were 0.019 and 0.027 for 100mM glucose and CHP, respectively. For pRb, *p* values were 0.037, 0.049, 0.001 and 0.002 for 100 mM glucose, CHP, CHL and GA, respectively. For pcdc2, *p* values were 0.011 and 0.042 for CHL and GA, respectively.

Cell cycle regulation is of critical importance in the senescence process. The cell cycle diagram and the cell numbers carried out in a flow cytometer showed that high glucose may cause cell senescence by interfering cell cycle progression. The extracts could affect high glucose. To confirm this, we investigated the expression of three proteins: cyclin D1 (G1 cyclin regulatory partner), pRb (G1 checkpoint) and pcdc2 (S and G2 checkpoint) using the Western blot. Figure 8, shows the expression of cyclin D1 and pRb was significantly increased when cells were treated with 100 mM of glucose alone (* *p* < 0.05 vs. control). When treated with 1 µg/mL of either CHL or GA, both pRb and pcdc2 proteins were significantly decreased and only cyclin D1 expression was reduced in response to treatment with 1 µg/mL CHP (** *p* < 0.05 vs. 100 mM glucose alone).

**Figure 9:**
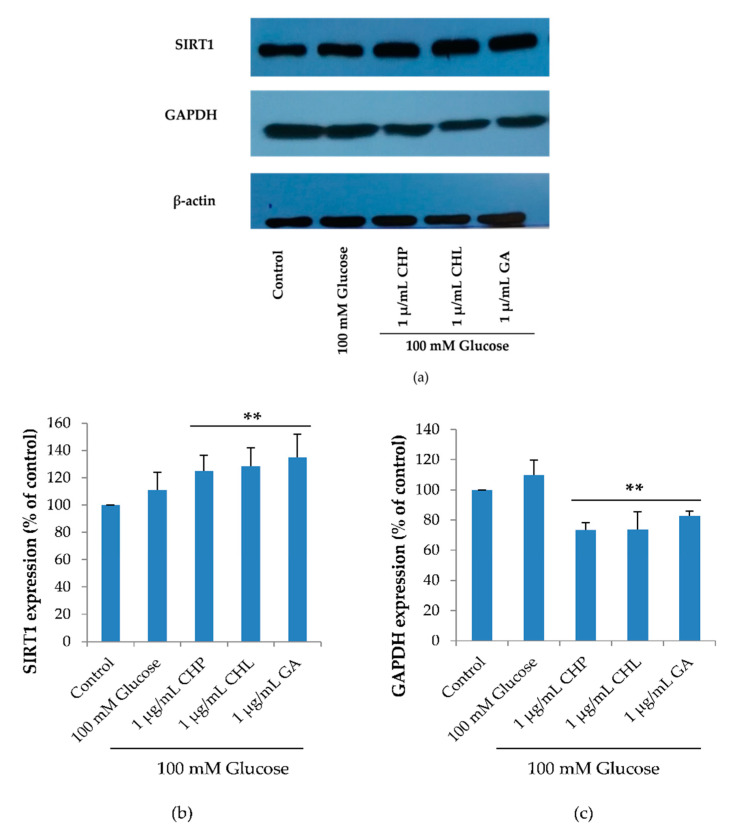
Surtuin 1 (SIRT1) and Glyceraldehyde 3-phosphate dehydrogenase (GAPDH) expression, as shown in a representative Western blot (**a**). Normalized values of SIRT1 and GAPDH against β-actin ((**b**) and (**c**))**.** The mean ± SD values of normalized SIRT1 and GAPDH expression were obtained from three independent experiments, * *p* < 0.05 vs. control; ** *p* < 0.05 vs. 100 mM glucose alone. For SIRT1, *p* values were 0.005, 0.011 and 0.005 for CHP, CHL and GA, respectively. For GAPDH, *p* values were 0.001, 0.001 and 0.008 for CHP, CHL and GA, respectively.

SIRT1 plays an important role in cell cycle and cell senescence. SIRT1 expression is a response to glucose starvation, as it activates GAPDH phosphorylation. As revealed in Figure 9, SIRT1 and GAPDH expression levels were not significantly different between the control and 100 mM glucose-treated groups. In extract-treated groups, CHP, CHL and GA significantly reduced glucose level in cells. When the glucose level was low, GAPDH was phosphorylated and phospho-GAPDH translocated into a nucleus that up-regulated SIRT1 expression. For these reasons, we found that SIRT1 expression significantly increased and decreased GAPDH expression, which was observed in extract-treated cells (** *p* < 0.05 vs. 100 mM glucose alone).

## 3. Discussion

*Citrus hystrix* is a medicinal plant that treats many ailments and it is used in many traditional medicines. Based on DPPH and ABTS assays, high antioxidant properties of its extracts were reported. Furthermore, the phytochemical constituents were analyzed and identified by GC-MS. The spectrum of GC-MS confirmed that CHP and CHL contain a number of various bioactive compounds. The nature and beneficial effects on health of these compounds were discussed in Table 6.

Hyperglycemia is a major cause of diabetes-associated neuronal diseases through cellular senescence. Cellular senescence is the process of an irreversible cell cycle arrest through many mechanisms, that is, inflammation cytokine, oxidative stress and cell cycle checkpoint [104,105,106]. Further, it causes many age-related chronic diseases such as atherosclerosis, Alzheimer’s disease and cancer [107,108,109,110,111]. The antidiabetic and antioxidant activity of CH and its phytochemical constituents was described earlier in human adipocytes, cataract in streptozotocin-induced diabetic rats [112] and hepatoprotective effect in paracetamol-induced injury in rats [23]. However, the exact pathway of protective effect of CH on high glucose-induced neuronal senescence was not explored.

Interestingly, CHP and CHL extracts appeared to effectively reduce the intracellular ROS accumulation at the concentration of 1 µg/mL but not at higher concentrations. Notably, it is interesting that CHP and CHL may also possess a pro-oxidant activity when administered at high doses. It is worth noting that contributing factors such as dosage, phytochemical profile and oxidation-reduction potential (ORP) may relate to the balance between the beneficial and deleterious activities [113]. The detail of ORP is described herein biochemical perspective. 

Given the effectiveness of both CHP and CHL to decrease ROS induced by high glucose, we hypothesized that both extracts may provide neuronal cell protection against cell cycle arrest induced by high glucose. To test our hypothesis, we first analyzed cell cycle via a flow cytometer.

For flow cytometry results (Figure 7a,b), the percentage of cells at the G1 phase in the 100 mM glucose-treated group significantly increased compared with the control group. In addition, both CHP and CHL extracts significantly decreased the percentage of the cell number in the G1 phase.

To further confirm the effect of CHP and CHL on cell cycle, the expression of cell cycle checkpoint proteins (pRb and cdc2), cyclin D1 and SIRT1 was detected by the Western blot.

**Figure 10:**
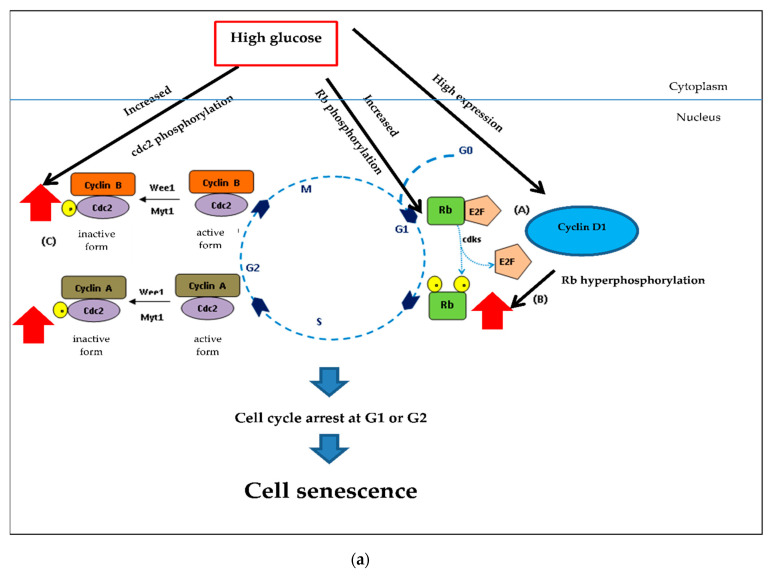

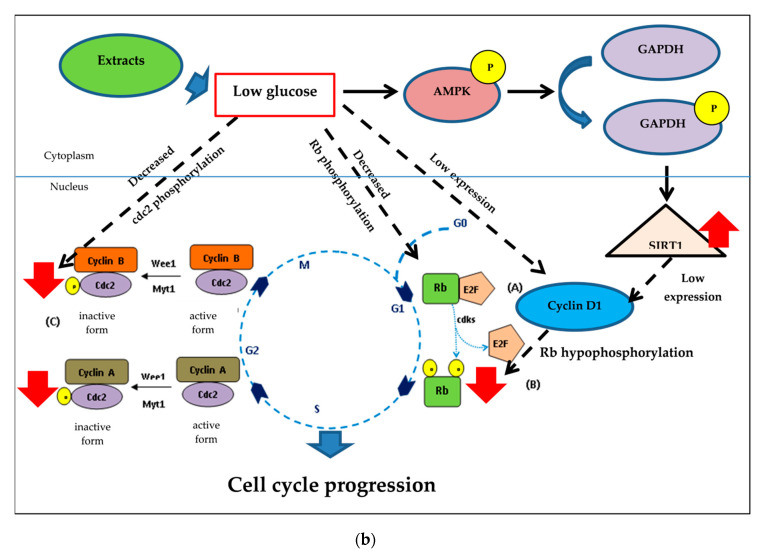
Underlying mechanisms of CHP and CHL exerts and their anti-senescent activity in SH-SY5Y cells. (**a**) In hyperglycemia, hyperphosphorylation of Rb induced by high glucose and cyclin D1 caused cell cycle arrest at G1. Hyperphosphorylated Rb failed to assemble with E2F. Furthermore, pcdc2 was an inactive form that caused cell cycle arrest. (**b**) Both extracts could reduce high glucose level. Low cellular glucose significantly increased GAPDH phosphorylation via AMP-activated protein kinase (AMPK), triggering SIRT1 activation. This mechanism induced the cell cycle progression by decreasing Rb and cdc2 phosphorylation. High expression of proteins (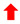); Low expression of proteins (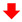); Hyperphosphorylation of proteins (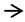); Hypophosphorylation of proteins (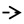).

Checkpoint processes control the cell cycle to avoid the accumulation of genetic damage. It does so by monitoring DNA integrity and cell growth during G1/S and G2/M transitions. These processes are common to all eukaryotic cells [114]. As Figure 10 shows, in the early and middle points of the G1 phase, Rb binds to E2F causing cells to arrest. However, in late stage of the G1 phase, Rb is phosphorylated by CDKs to increase gene transcription and permit cells to enter the S phase. Cyclin/cdc2 complexes control the cell cycle. Cdc2 is high in the S and G2 phases. In the S phase, G2/M transition control binds to cyclin A and cyclin B, respectively. Cyclin/cdc2 complexes are inactive due to Wee1/Myt1 phosphorylation.

The Western blot results demonstrated that high glucose significantly increased cyclin D1, pRb and pcdc2. Increased cyclin D1 could induce phosphorylation of Rb (Figure 10a). Although the pRb/E2F complex permits cell progression in the G1 and S phase, in hyperglycemia, hyperphosphorylation of Rb-induced high glucose and cyclin D1 caused cell cycle arrest at G1. Many studies have indicated that hyperphosphorylated Rb failed to assemble E2F [115,116,117]. Furthermore, we found pcdc2 to be an inactive form that caused cell cycle arrest.

Although both CHP and CHL were similarly effective in reducing ROS accumulation, they exerted neuronal cell protection against cell cycle arrest induced by high glucose through different signaling pathways. We observed that CHP significantly decreased only cyclin D1 expression in extract-treated groups, while CHL significantly decreased both pRb and pcdc2 expression. However, taken together, our results showed that both CHP and CHL could provide neuronal cell protection against cell cycle arrest induced by high glucose.

Noticeably, the difference of the effect on cell-cycle associated protein expression between CHP-treated and CHL-treated groups might be due to the compositional differences of the two extracts, as shown by the GC-MS analysis. GC-MS analysis (Table 4 and Table 6) revealed that phytochemical constituents of isooxypeucedanin, α-terpineol and umbelliferone were only found in CHP and exerted antidiabetic effect by normalizing blood glucose level and re-establishing insulin sensitivity [57,58,66]. The previous studies showed the effect of certain antidiabetic drugs on the cell-cycle through the decrease of cyclin D1 expression [118]. Cyclin D1 is an essential link between cell-cycle and energy control metabolism [119]; this may explain why CHP was only effective in reducing cyclin D1 protein expression but not pRb and pcdc2 expression.

SIRT1 is an important protein that plays a role in cell senescence. We hypothesized that both CHP and CHL could protect cell senescence by up-regulating SIRT1 expression. The Western blot results confirmed our hypothesis. When cells were treated with either CHP or CHL, SIRT1 expression was significantly up-regulated.

Furthermore, we hypothesized that SIRT1 would up-regulate through the phosphorylation of GAPDH. GAPDH and SIRT1 expression, when treated with either CHP or CHL, confirmed that both extracts could reduce the glucose level in cells. Figure 10b showed the underlying mechanisms of both extracts. Low cellular glucose significantly increased the phosphorylation of GAPDH and SIRT1 expression. When cellular glucose decreased, GAPDH became a critical enzyme and was phosphorylated by AMP-activated protein kinase (AMPK), thus triggering SIRT1 activation. This process was necessary to protect cells from glucose starvation, such as autophagy and gluconeogenesis [120]. Results from the Western blot showed the association of SIRT1 and cyclin. These results are consistent previous studies, that is, down-regulated SIRT1 was arrested in the G1 phase via cyclin D1 signaling [121,122].

Thus, all results showed that both CHP and CHL could attenuate high glucose-induced cellular senescence in human neuronal SH-SY5Y cells by inducing the cell cycle progression and up-regulation of SIRT1.

In this study, we found CH extracts with high ROS-scavenging properties and they were able to protect high glucose-induced human neuronal senescence. The antioxidant and anti-senescent properties of CH extracts are also extensively discussed in further characterization from two perspectives.

### 3.1. A Biochemical Perspective

Notably, in this study, radical scavenging activity assays of CHP and CHL extracts dissolved in DMSO were performed with DPPH and ABTS in ethanol. However, it still needs to be established whether CHP and CHL extracts’ radical scavenging activities could persist in living tissues’ aqueous environment under physiological conditions. CH extracts should be administered to animals treated with high glucose to induce senescence. Followed by CH’s biochemical evaluation on oxidative stress in blood and tissue samples should be investigated to clarify whether CHP and CHL have scavenging activities under physiological conditions. For example, using samples derived from blood or tissues, antioxidant biomarkers (such as Glutathione (GSH)) could be analyzed. The GSH content indicates the cell’s defense against ROS causing cellular injury [123,124] and reflecting the ability of a tissue to scavenge excess superoxide anions leading to oxidative stress [125], Malondialdehyde (MDA)-a direct indicator of cell membrane damage occurring in the tissue [126,127], Catalase (CAT)-an indicator of CH toleration by that particular tissue [128], Superoxide dismutase (SOD)-an indicator of the active enzyme involvement in neutralizing the effect of free radicals [129] and Level of protein content-an indicator of cell capacity of mitigating the effect of free radical and peroxide processes resulting in modulating the cellular antioxidant status [130]. Furthermore, other aspects, as below, are also concerned as described below [131,132].

**Figure 11:**
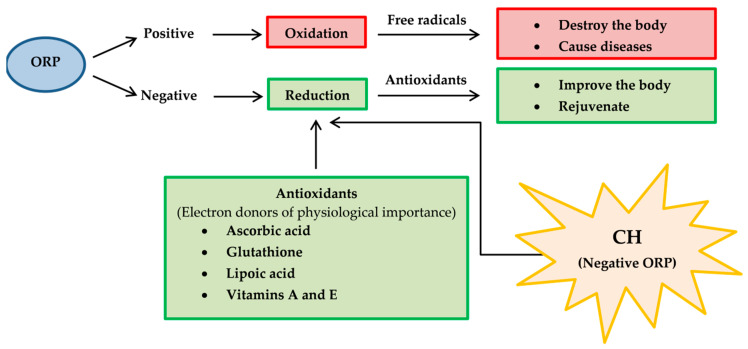
The potential applications of oxidation-reduction potential (ORP) or redox reaction for CH determination in pharmaceuticals.

- Therapeutic agents with high lipid solubility can penetrate cells more rapidly than therapeutic agents with water solubility. So, lipid or water solubility should be assessed.
- Most of the therapeutic agents are available as weak acids or weak bases. Thus the pH of therapeutic agents should be addressed appropriately.
- Oxidation-reduction potential (ORP) [133], also known as redox, should be determined because it reflects a molecule’s ability to oxidize or reduce another molecule. Reducers have negative ORP and oxidizers have positive ORP. Typically, all organs in our body have negative OPR (−10 to −250 mV of ORP). Oxidizers can oxidize another molecule, which causes it to lose electrons. Oxidizers become free radicals after accepting radicals and cause many diseases. On the other hand, free radicals can be neutralized with reducers and antioxidants, causing body improvement and rejuvenation. For medicinal benefits, CH or their major active constituents should have negative ORP and play a role as a reducer or antioxidant. Therefore, the proposed application of redox reactions is useful for CH determination in pharmaceuticals (Figure 11).
- Oxygen partial pressure (PO_2_) [134]: PO_2_ is the force exerted by oxygen. In the human body (highly aerobic organism), oxygen plays a role in energy production. Therefore, oxygen supply at the tissue must match metabolic demand. PO_2_ is useful to maintain homeostasis (the balance between oxygen delivery and its consumption) within organ and tissue. Each organ and tissue has its PO_2_ requirements in order to function correctly. PO_2_ is useful in predicting oxygen and oxygen movement will move from a higher PO_2_ area to a lower PO_2_ area. Typically, PO_2_ in tissue is low because oxygen is used in cellular respiration. When PO_2_ in tissue increased by several factors such as stress, anesthesia, tumor and diabetes, oxygen availability is low or hypoxia. In consideration of CH’s pharmacology, the importance of hypoxia as below is concerned [135].

1. Hypoxia may alter the therapeutic effectiveness and metabolism of CH.
2. Hypoxia may alter cellular function.
3. Hypoxia may potentiate or mitigate CH-induced toxicity.
4. CH may potentiate or protect against hypoxia-induced pathology.
5. CH may alter the relative coupling of blood flow and energy metabolism in an organ.

### 3.2. A Pharmacokinetic Perspective

**Figure 12:**
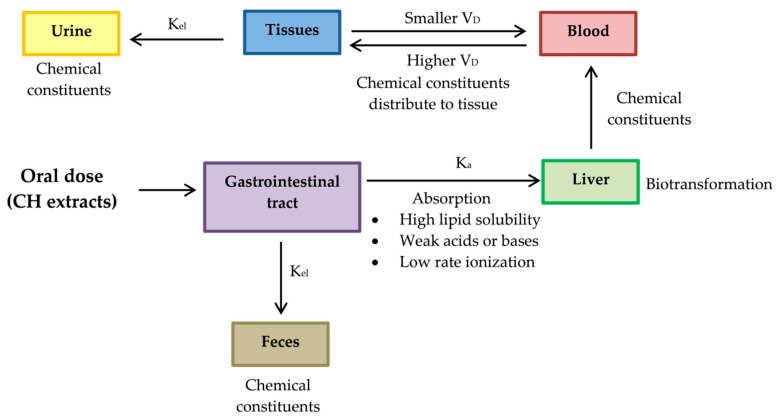
The proposed diagram of pharmacokinetics for evaluating of active phytochemicals of CH extracts. After chemical constituents are absorbed by gastrointestinal tract, they are delivered to liver, where they are biotransformed and delivered into blood. Chemical constituents are biodistributed to other peripheral organs by blood stream. Absorbed chemical constituents are excreted through the urine but certain chemical constituents that are not absorbed through the gastrointestinal tract will be eliminated through feces. Absorption rate constant (*K*_a_); Constant elimination (*K*_el_); Volume of distribution (*V*_D_).

It is universally agreed before any novel bioactive compound considered a lead for developing an active principle of any preventive or therapeutic usefulness, it is essential to conduct exhaustive animal and pre-clinical studies. Therefore, pharmacokinetic evaluations of CH extracts’ active phytochemicals should be studied in animal models including, blood samples for absorption studies, tissues such as intestine, liver, lungs, kidney and heart distribution studies and urine and excreta collection for excretion studies. After sample preparation, extraction of bioactive markers and assessment of pharmacokinetic parameters as listed below should be carried out [136]. The proposed pharmacokinetic diagram after administering the active phytochemical of CH extracts in an animal model is shown in Figure 12.

- Absorption rate constant (*K*_a_) should be determined for a chemical compound of CH. The compound investigated should have high *K*_a_, so its characteristics should be high lipid solubility, weak acids or weak bases and low rate ionization.
- Constant elimination (*K*_el_) is a value that describes the rate at which an active compound of CH is removed from the human system. Its value is affected by all processes such as distribution, biotransformation and excretion.
- Volume of distribution (*V*_D_) represents the distribution of a compound of CH in body tissues rather than the plasma. If *V*_D_ is higher than the total body water, it indicates a greater amount of tissue distribution. A smaller *V*_D_ means a compound remains in the plasma than CH distribution in tissues [137]. A compound studied for medicinal benefits should have higher *V*_D_, so it should be characterized by high lipid solubility, low rates of ionization and low plasma protein binding capabilities.
- Biotransformation represents the chemical alteration process of therapeutic agents in the body.

As far as pharmacokinetics is concerned, individual chemical constituents found in CH were previously studied, such as sitosterol [138] and caryophyllene [139].

## 4. Materials and Methods

### 4.1. Chemicals and Reagents

Dimethyl sulfoxide (DMSO) and ethanol were purchased from Merck (Darmstadt, Germany). Phenylmethyl sulphonyl fluoride (PMSF) was purchased from United States Biological (Cleveland, OH, USA). Kodak processing chemicals were used for autoradiography films. The Amersham ECL select Western blotting detection reagent was purchased from GE Healthcare (Piscataway, NJ, USA). Dulbecco’s modified Eagle medium (DMEM)/low glucose, fetal bovine serum (FBS) and penicillin-streptomycin solution (10,000 units/mL of penicillin and 10,000 μg/mL of streptomycin) were purchased from HyClone (Logan, UT, USA). A solution of 30% acrylamide/bis-acrylamide (37.5:1) was purchased from Biorad (Hercules, CA, USA). Ammonium persulfate (APS) was purchased from EMD Millipore (Billerica, MA, USA). The monoclonal rabbit SIRT1 (D1D7, cat#9475), GAPDH (14C10, cat#2118), Cyclin D1 (92G2, cat#2978), Phospho-Rb (Ser807/811, cat#9308), Phospho-cdc2 (Tyr15, cat#9111) and β actin (13E5, cat#4970) were purchased from Cell Signaling Technology (Beverly, MA, USA). Propidium iodide (PI) was purchased from Biolegend (San Diego, CA, USA).

### 4.2. Plant Extraction

*Citrus hystrix* (or kaffir lime) peels and leaves were designated as CHP and CHL, respectively and were cultivated in the Bang Ramat District, Taling Chan County and Bangkok, Thailand (Latitude, Longitude: 13.777883, 100.416748). They were harvested in October and November 2019. They were extracted by maceration method using 70% ethanol (ratio 1:5 *w*/*v*) at room temperature (RT) in the dark for 48 h and filtered. The residue was extracted twice. The two filtrates were combined and concentrated by evaporation at 45 ºC. The crude extracts were dissolved in DMSO or kept at −80 °C until further investigation.

### 4.3. Gas Chromatograph–Mass Spectrometer (GC-MS) Analysis

The extracts were submitted to the Scientific and Technological Research Equipment Center (STREC) (Chulalongkorn University, Thailand). The GC-MS Triple Quad system was an Agilent 7890 series GC system coupled with an Agilent 7000C MS and a capillary column (HP-5MS 5% Phenyl Methyl Siloxane, length 30 m, i.d. 0.25 mm, phase thickness 0.25 µm). The GC was operated with helium as the carrier gas (1 mL/min). The inlet had a temperature of 250 °C, pressure set to 8.2317 psi and 1.5 µL injection. The GC oven was kept at 60 °C for 3 min before rising to 325 °C (with linear gradient of 5 °C/min) and kept at 325 °C for 3 min. The total run time was 14 min. The extracts (~10 mg) were dissolved in 1 mL of absolute ethanol and their obtained spectra were compared with NIST Mass Spectrometry Data Center to identify phytochemical constituents.

### 4.4. Antioxidant Determination

#### 4.4.1. Folin–Ciocalteu Phenol Assay (FCP)

The extracts (50 µL) and 10% Folin–Ciocalteu Phenol reagent (50 µL) were mixed and incubated in the dark at room temperature (RT) for 30 min. A sodium carbonate (Na_2_CO_3_) solution (35 µL) was added, mixed and incubated in the dark at RT for 20 min. Reaction absorbance was measured using the Enspire^®^ Multimode Plate Reader (Perkin-Elmer) at 750 nm. Gallic acid was used as the standard. The amount of phenolic compound was in a Gallic acid equivalent (GE) mg/g of dry weight.

#### 4.4.2. Total Flavonoid of Determination

The extracts (50 µL) were mixed with the solution (150 µL of ethanol, 10 µL of 1M Sodium acetate (NaOAc) and 10 µL of Aluminum Chloride (AlCl_3_)). The mixture was incubated in the dark at RT for 40 min and measured at 415 nm. Quercetin was used as the standard. The content of flavonoid was in Quercetin equivalent (QE) mg/g of dry weight.

#### 4.4.3. Radical Scavenging Activity Assays

Next, 0.2 mg/mL of 2,2-diphenyl-1-picryl-hydrazyl-hydrate (DPPH^•^) and freshly prepared 2,2′-azino-bis (3-ethylbenzthiazoline-6-sulphonic acid) (ABTS^•+^) (OD_734_ = 0.7–0.8) were diluted in ethanol. The extract (1 mg/mL) was reacted with DPPH^•^ or ABTS^•+^ and incubated at RT for 15 and 30 min, respectively. Absorbance was measured at 517 nm and 734 nm, respectively. Ascorbic acid (Vitamin C) was used as the standard for both assays. The antioxidant capacity had Vitamin C equivalent antioxidant capacity (VCEAC) in mg/g of dry weight.

### 4.5. Cell Line

SH-SY5Y cells—a human neuroblastoma cell line—were purchased from a cell line service (Heidelberg, Germany; Catalogue number 300154). They were cultured in DMEM/low glucose (HyClone, USA) containing 10% FBS and antibiotics (100 U/mL penicillin and 100 μg/mL streptomycin) at 37 °C in a humidified atmosphere at 5% CO_2_.

### 4.6. 3-(4,5-Dimethylthiazol-2-yl)-2,5-diphenyltetrazolium bromide tetrazolium (MTT) Assay

We determined the nontoxic concentration of CHP and CHL extracts with SH-SY5Y. Cells were seeded at 20,000 cells/well plates and incubated at 37 °C for 24 h. Cells were treated with various concentrations of extracts for 24 h. MTT (5 mg/mL) was added to each well (20 µL/well) and incubated for 4 h. Media was removed carefully. In this step, a formazan product was formed and dissolved with 150 µl of 100% DMSO. A supernatant was collected and transferred to a new 96-well plate. Moreover, it measured the absorbance with a spectrometer at 550 nm. The nontoxic concentration of the extracts was shown as the percentage of cell viability calculated by the following formula.

$$\%\;\mathrm{cell}\;\mathrm{viability}=\frac{\;\left({\mathrm{Abs}}_{\mathrm{treated}\;\mathrm{cells}}–{\mathrm{Abs}}_{\mathrm{blank}}\;\right)\;\times \;100}{{\mathrm{Abs}}_{\mathrm{untreated}\;\mathrm{cells}}–{\mathrm{Abs}}_{\mathrm{blank}}}$$

### 4.7. Reactive Oxygen Species (ROS) Assay

The appropriate concentration of extracts was tested. Cells were seeded at 20,000 cells/well in 96-well plates and incubated at 37 °C for 24 h. Cells were treated or co-treated with 100 mM glucose or extracts for 24 h. Next, 5 µM of non-fluorescent 2′,7′-dichloro-dihydrofluorescein diacetate (H_2_DCFDA) was loaded, incubated at 37 °C for 45 min and then washed 3 times with PBS. The level of intracellular ROS was measured based on the ability of ROS to oxidize non-fluorescent H_2_DCFDA into a highly fluorescent 2′,7′-dichlorofluorescein (DCF). The fluorescence was measured with an excitation wavelength of 485 nm and an emission wavelength of 535 nm.

### 4.8. Cell Cycle Assay by Flow Cytometer

Cells were seeded at 500,000 cells/well in 6-well plates and incubated at 37 °C for 24 h. Having been incubated, cells were co-treated with 100 mM glucose and 1 µg/mL of CHP, CHL or gallic acid for 24 h. After treatment, cells were harvested, washed in cold PBS and re-centrifuged at 400 g for 5 min. Cells were re-suspended in absolute ethanol at −20 °C for at least 2 h. Cells were washed and re-suspended in 1% (*v*/*v*) Triton X-100 PBS and treated with RNase. Next, 500 µL of PI/Triton X-100 staining solution was added and incubated at 37 °C for 15 min. The cell cycle was analyzed via flow cytometry (FACSCalibur (BD Biosciences, San Jose, CA, USA)).

### 4.9. Protein Expression by Western Blotting

Cells were seeded at 500,000 cells/well in 6-well plates and incubated at 37 °C for 24 h. Cells were co-treated for 24 h. The next day, protein extraction was carried out using 1 mM of PMSF in a NP-40 lysis buffer. Total protein (40 μg) was mixed with a 2× Laemmli buffer (ratio 1:1) and heated at 95 °C for 10 min. Protein was separated with 10% sodium dodecyl sulfate-polyacrylamide gel electrophoresis (SDS-PAGE) and transferred onto polyvinylidene difluoride (PVDF) membranes. Membranes were blocked with 5% nonfat milk for 1 h at room temperature. Membranes were incubated with primary antibodies (cyclin D1 (1:2000), pRb (1:2000), pcdc2 (1:2000), SIRT 1 (1:2000), GAPDH (1:10,000) and β actin (1:2000)) overnight at 4 °C. After incubation, membranes were washed 3 times with 1× TBS-Tween 20 (TBST) for 15 min, incubated with secondary antibodies (anti-rabbit IgG, HRP-linked antibody) for 45 min at RT and washed 3 times with TBST for 15 min. Protein bands were visualized by adding an enhanced chemiluminescence detection reagent using autoradiography films and Kodak processing chemicals. Each band was normalized against β actin as an internal control.

### 4.10. Statistical Analysis

Data were presented as the mean ± standard deviation (SD). Means were from at least three independent experiments. Data were analyzed via a one-way analysis of variance (ANOVA) followed by a post hoc Tukey test (*p* value ˂ 0.05) using low glucose-treated cells as the control group.

## 5. Conclusions

In summary, our results demonstrate that CH is an interesting plant with rich antioxidant properties and bioactive compounds. Both CHP and CHL can protect human neuronal cells from glucose-induced neuronal senescence. The neuroprotective effect of CHP and CHL is mediated through cell cycle progression in cell cycle checkpoint proteins and SIRT1 up-regulation after SIRT1/GAPDH pathway activation. CH extracts could be developed as agents for the protection of high glucose-induced neuronal senescence.

However, the bioactivities of this extract needs to be further explored in other living organisms. Hopefully, neuronal senescence-associated diseases will be clarified in further investigations.

## Figures and Tables

**Table 1:** Free radical scavenging activities of ethanolic extracts of *Citrus hystrix* (CH) using a 2,2-diphenyl-1-picryl-hydrazyl-hydrate (DPPH) scavenging assay.

Sample	% Radical Scavenging Activity (of 1 mg/mL Extract)	mg VCEAC/g Dry Weight Sample
CHP	14.98 ± 5.25	210.06 ± 11.95
CHL	15.36 ± 6.79	238.89 ± 12.25

VCEAC: Vitamin C equivalent antioxidant capacity.

**Table 2:** Free radical scavenging activities of ethanolic extracts of CH using a 2,2′-azino-bis (3-ethylbenzthiazoline-6-sulphonic acid) (ABTS) scavenging assay.

Sample	% Radical Scavenging Activity (of 1 mg/mL extract)	mg VCEAC/g Dry Weight Sample
CHP	90.38 ± 0.11	3063.67 ± 3.71
CHL	65.18 ± 0.33	2219.99 ± 11.12

VCEAC: Vitamin C equivalent antioxidant capacity.

**Table 3:** Total phenolic and flavonoid contents of ethanolic extracts of CH.

Sample	Total Phenolic (mg(GA)/g of Dry Weight)	Total Flavonoid (mg(QE)/g of Dry Weight)
CHP	1796.55 ± 1.38	1521.54 ± 3.54
CHL	2134.48 ± 1.06	2856.15 ± 1.24

GA: Gallic acid; QE: Quercetin.

**Table 4:** Proposed phytochemical constituents in *Citrus hystrix* peels (CHP) compared with the National Institute of Standards and Technology (NIST) database.

Peak No.	RT	Area (%)	MF	MW	Name of Compound
12	12.635	0.9	C_10_H_18_O	154	Citronellal
15	13.735	0.87	C_10_H_18_O	154	α-Terpineol
18	14.765	1.01	C_10_H_20_O	156	Citronellol
23	16.061	1.08	C_8_H_14_O_3_	158	Methyl 6-oxoheptanoate
30	18.872	0.83	C_15_H_24_	204	α-Copaene
36	20.018	0.58	C_15_H_24_	204	Caryophyllene
40	21.559	1.13	C_15_H_24_	204	β-Cubebene
46	22.558	3.54	C_15_H_24_	204	Cadinene
44	22.2	0.24	C_14_H_22_O	206	Phenol, 2,4-bis(1,1-dimethylethyl)-
61	29.504	0.7	C_9_H_6_O_3_	162	7-Hydroxycoumarin
62	31.821	1.23	C_16_H_32_O_2_	256	n-Hexadecanoic acid
63	32.495	0.39	C_18_H_36_O_2_	284	Hexadecanoic acid, ethyl ester
68	34.698	0.41	C_20_H_40_O	296	Phytol
76	36.32	6.86	C_11_H_6_O_4_	202	7*H*-Furo(3,2-g)(1)benzopyran-7-one, 9-hydroxy-
77	40.871	14.86	C_16_H_14_O_5_	286	7*H*-Furo(3,2-g)(1)benzopyran-7-one, 4-(2,3-epoxy-3-methylbutoxy)-, (*S*)-(−)-
78	41.405	1.37	C_16_H_14_O_5_	286	4-(3-Methyl-2-oxobutoxy)-7*H*-furo(3,2-g)(1)benzopyran-7-one
82	44.565	12.4	C_16_H_16_O_6_	304	4-(2,3-Dihydroxy-3-methylbutoxy)furo(3,2-g)chromen-7-one
88	52.412	0.81	C_29_H_50_O	414	Sitosterol

RT: retention time; MF: molecular formula; MW: molecular weight.

**Table 5:** Proposed phytochemical constituents in *Citrus hystrix* leaves (CHL) compared with the NIST database.

Peak No.	RT	Area (%)	MF	MW	Name of Compound
9	12.631	2.35	C_10_H_18_O	154	Citronellal
13	14.758	3.53	C_10_H_20_O	156	Citronellol
17	17.664	4.24	C_10_H_20_O_2_	172	Cyclohexanol, 2-(2-hydroxy-2-propyl)-5-methyl-
28	20.014	3.51	C_15_H_24_	204	Caryophyllene
36	22.193	0.78	C_14_H_22_O	206	Phenol, 2,4-bis(1,1-dimethylethyl)-
40	23.447	2.39	C_15_H_26_O	222	1,6,10-Dodecatrien-3-ol, 3,7,11-trimethyl-
55	31.918	0.19	C_20_H_30_O_4_	334	1,2-Benzenedicarboxylic acid, butyl octyl ester
57	32.494	2.52	C_18_H_36_O_2_	284	Hexadecanoic acid, ethyl ester
61	34.701	17.3	C_20_H_40_O	296	Phytol
65	40.803	1.53	C_16_H_14_O_5_	286	7*H*-Furo(3,2-g)(1)benzopyran-7-one, 4-(2,3-epoxy-3-methylbutoxy)-, (*S*)-(−)-
68	44.484	0.73	C_16_H_16_O_6_	304	4-(2,3-Dihydroxy-3-methylbutoxy)furo(3,2-g)chromen-7-one
71	52.408	4.91	C_29_H_50_O	414	Sitosterol

RT: retention time; MF: molecular formula; MW: molecular weight.

**Table 6:** Compound nature and bioactivity of phytochemical constituents in CHP and CHL.

Name of Compound	Compound Nature	Bioactivity
Citronellal	Monoterpenoid	Antibacterial and antifungal activities [24,40]
		Wound healing property on chronic diabetic wounds [27]
		Relaxing effects [61,62]
Citronellol	Monoterpene alcohol	Anti-inflammatory and analgesic activities [30,63]
Cyclohexanol, 2-(2-hydroxy-2-propyl)-5-methyl-	Monoterpenoid	Insect repellents [64,65]
Caryophyllene	Monoterpenes	Anti-inflammatory pathologies, atherosclerosis and tumors [35,66,67]
		Antioxidant activity [68,69]
		Analgesic activity [70,71]
2,4-bis(1,1-dimethylethyl)Phenol	Phenol	Antioxidant activity [72,73,74]
		Anti-inflammatory activity [74,75]
1,6,10-Dodecatrien-3-ol, 3,7,11-trimethyl- or (Nerolidol)	Sesquiterpene alcohol	Antioxidant activity [76,77,78,79,80] Anti-inflammatory and analgesic activities [81,82] Neuroprotective effect [36]
1,2-Benzenedicarboxylic acid, butyloctyl ester	Ester	Antioxidant activity [83]
Hexadecanoic acid, ethyl ester or (Ethyl palmitate)	Palmitic acid ester (Fatty acid ethyl ester)	Antioxidant, hypocholesterolemic, anti-androgenic [84] Anti-inflammatory activities [42]
Phytol	Diterpene alcohol	Antioxidant and neuroprotective effects [41,85]
7*H*-Furo(3,2-g)(1)benzopyran-7-one, 4-(2,3-epoxy-3-methylbutoxy)-, (*S*)-(−)- or (Heraclenin)	Furanocoumarin	Anti-inflammatory activity [86]
4-(2,3-Dihydroxy-3-methylbutoxy) furo(3,2-g)chromen-7-one or (Oxypeucedanin hydrate oraviprin)	Furanocoumarin	Antioxidant activity Anticancer activity [87,88]
Sitosterol	Phytosterol	Prevention of the coronary heart disease [43,44] Anti-Alzheimer’s activity [45]
		Antioxidant activity [46,47] Prevention of glutamate and β-amyloid toxicity [48]
α-Terpineol	Monoterpene alcohol	Antioxidant activity, antiulcer activity
		Cardiovascular and antihypertensive effects
		Anticonvulsant and sedative activity Re-establish insulin sensitivity Antibacterial activity Anti-nociceptive activity [49,50,51,52,53]
Methyl 6-oxoheptanoate	Methyl ester	Anticancer activity [89]
α-Copaene	Sesquiterpene	Antioxidant and anticancer activities [90,91]
Cadinene	Sesquiterpene	Antioxidant activity [92]
7-Hydroxycoumarin or Umbelliferone	Coumarin	Antihyperlipidemic and antidiabetic effects [93,94] Anti-inflammatory and antioxidant activities [95,96] Neuroprotective effect [97,98,99]
n-Hexadecanoic acid	Palmitic acid	Anti-inflammatory and antioxidant activities [84,100]
7*H*-Furo(3,2-g)(1)benzopyran-7-one, 9-hydroxy- or (Xanthotoxol)	Furanocoumarin	Antioxidant activity and neuroprotective effect [101,102]
4-(3-Methyl-2-oxobutoxy)-7*H*-furo(3,2-g)(1)benzopyran-7-one or (Isooxypeucedanin)	Furanocoumarin	Antidiabetic effects [103]
